# Supplementary material for: One-Step Multifunctionalization of Flax Fabrics for Simultaneous Flame-Retardant and Hydro-Oleophobic Properties Using Radiation-Induced Graft Polymerization
Source: Polymers (Basel). 2023 May 2;15(9):2169. doi: 10.3390/polym15092169 (PMC10180769; doi:10.3390/polym15092169)
Supplement: Supplementary file 1 [file polymers-15-02169-s001.zip › polymers-2331455-supplementary.pdf]

One-Step Multifunctionalization of Flax Fabrics for Simultaneous Flame-Retardant and Hydro-Oleophobic Properties Using Radiation-Induced Graft Polymerization

Table S1 : Molar masses, fluorine and phosphorus contents of the monomers used.

Monomer	Molar Mass ($g.mol^{-1}$)	Fluorine content (wt%)	Phosphorus content (wt%)
M4	332.2	51.5	-
AC6	418.0	59.1	-
M8	532.2	60.7	-
MAPC1	208.0	-	14.9

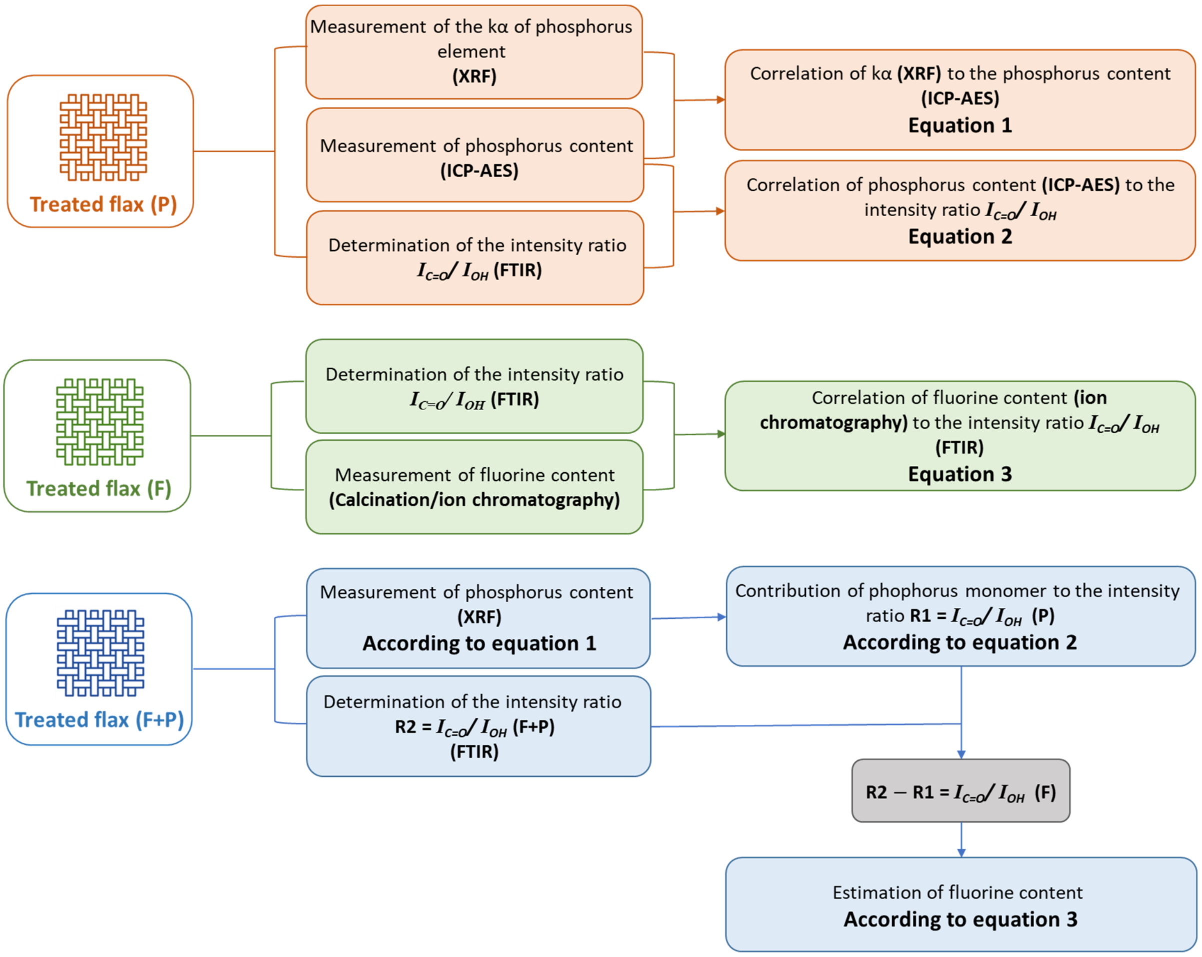

Scheme S1 : Multi-step procedure for calculation of the phosphorus and fluorine contents of modified flax fabrics.

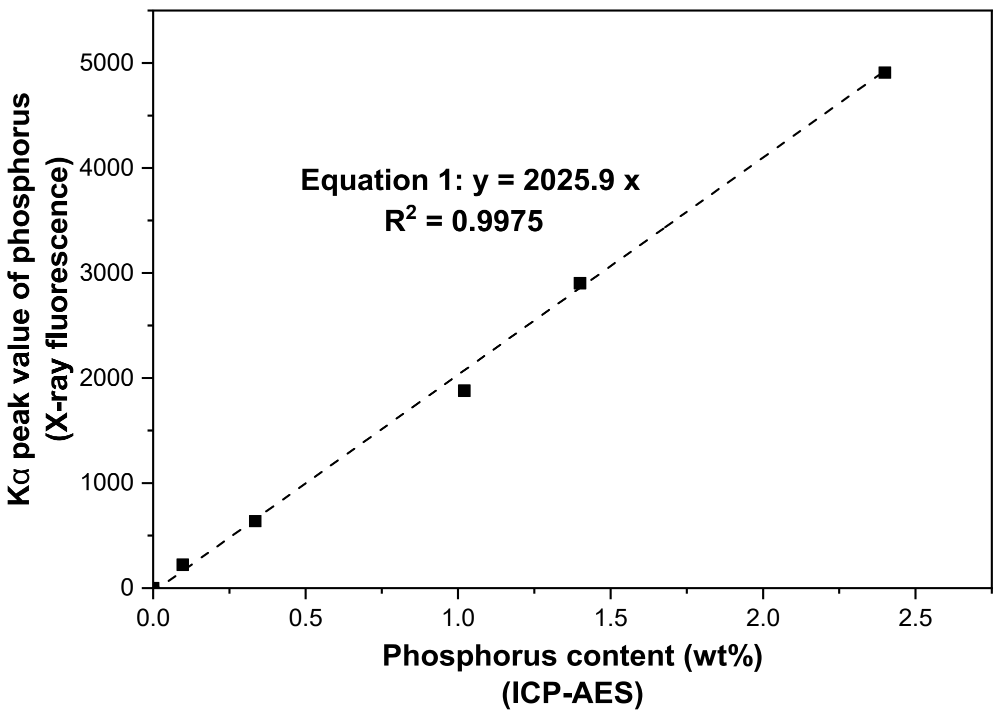

Figure S1 : Calibration curve of Kα peak value of phosphorus measured by X-ray fluorescence versus the phosphorus content determined by ICP-AES.

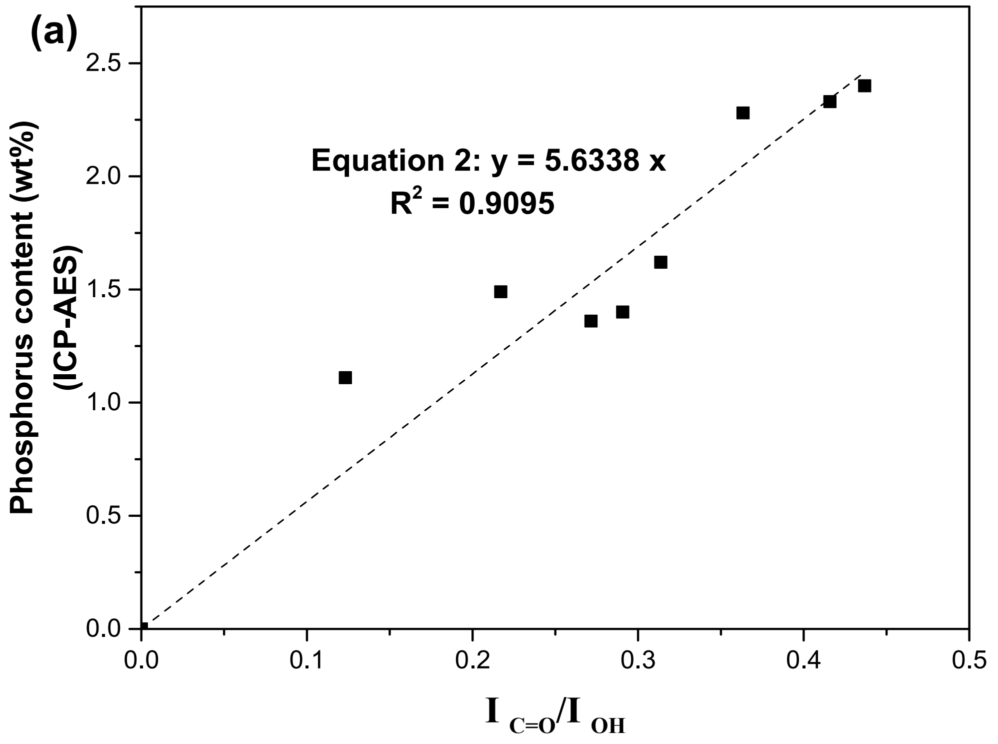

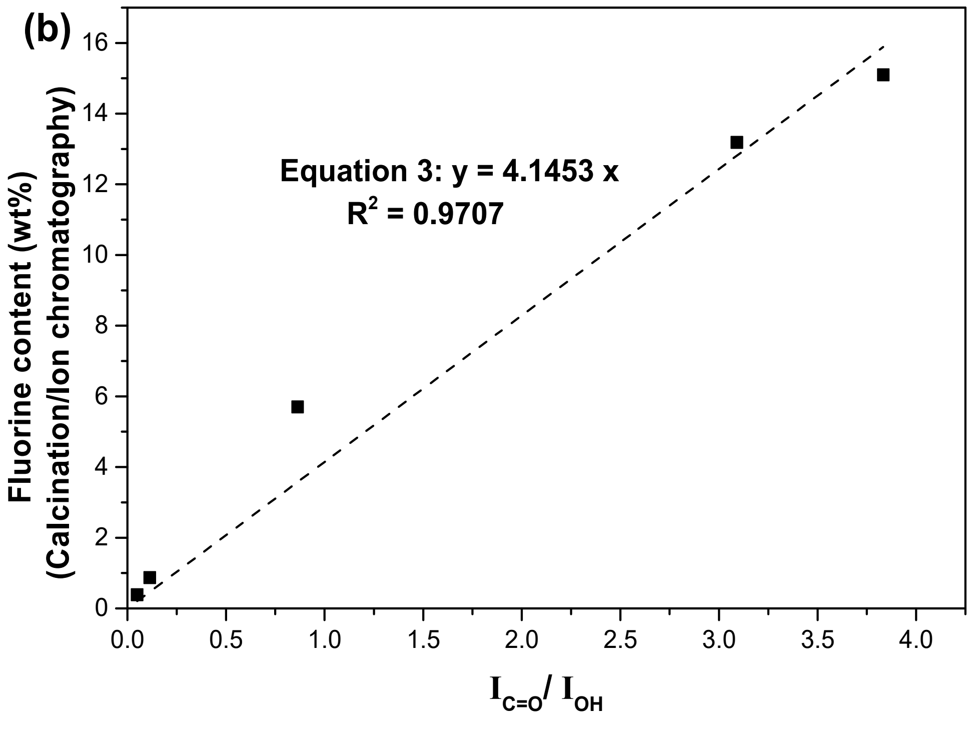

Figure S2 : Calibration curves of (a) phosphorus content determined by (ICP-AES) versus the intensity IC=O/ IOH ratio for samples treated only with MAPC1, (b) fluorine content measured by calcination followed by ion chromatography versus the intensity IC=O/ IOH ratio for samples treated only with M8.

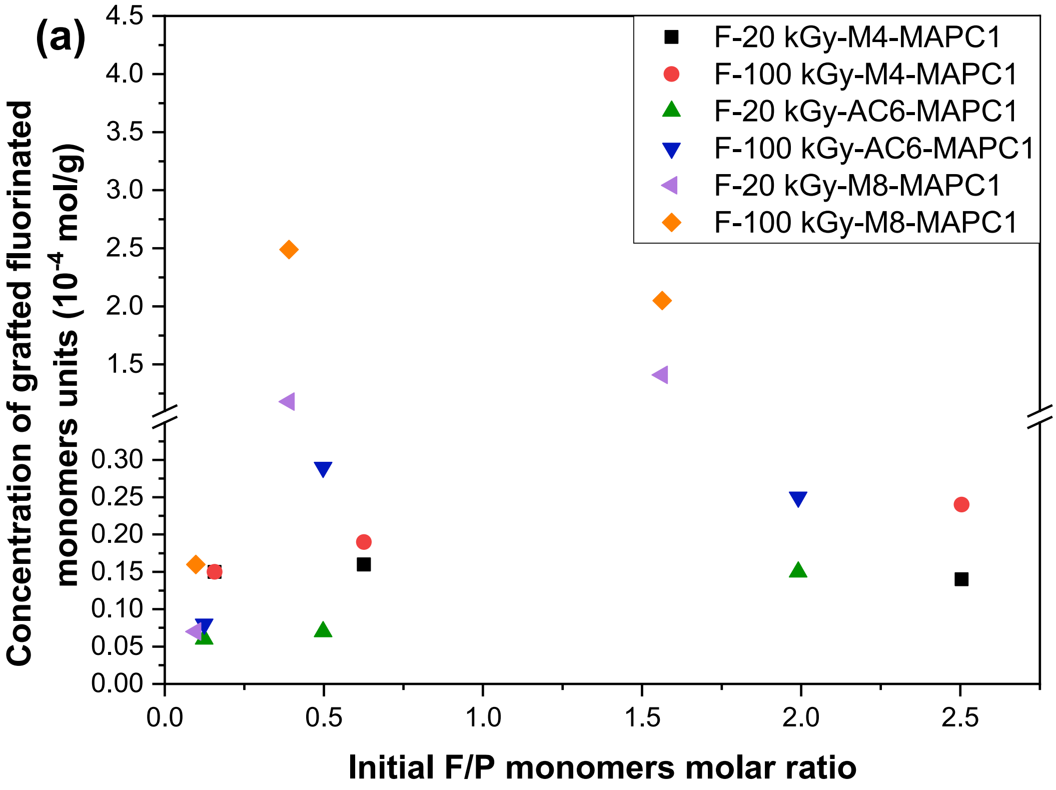
(a)
Concentration of grafted fluorinated monomers units (10^{-4} mol/g)
Initial F/P monomers molar ratio
F-20 kGy-M4-MAPC1
F-100 kGy-M4-MAPC1
F-20 kGy-AC6-MAPC1
F-100 kGy-AC6-MAPC1
F-20 kGy-M8-MAPC1
F-100 kGy-M8-MAPC1

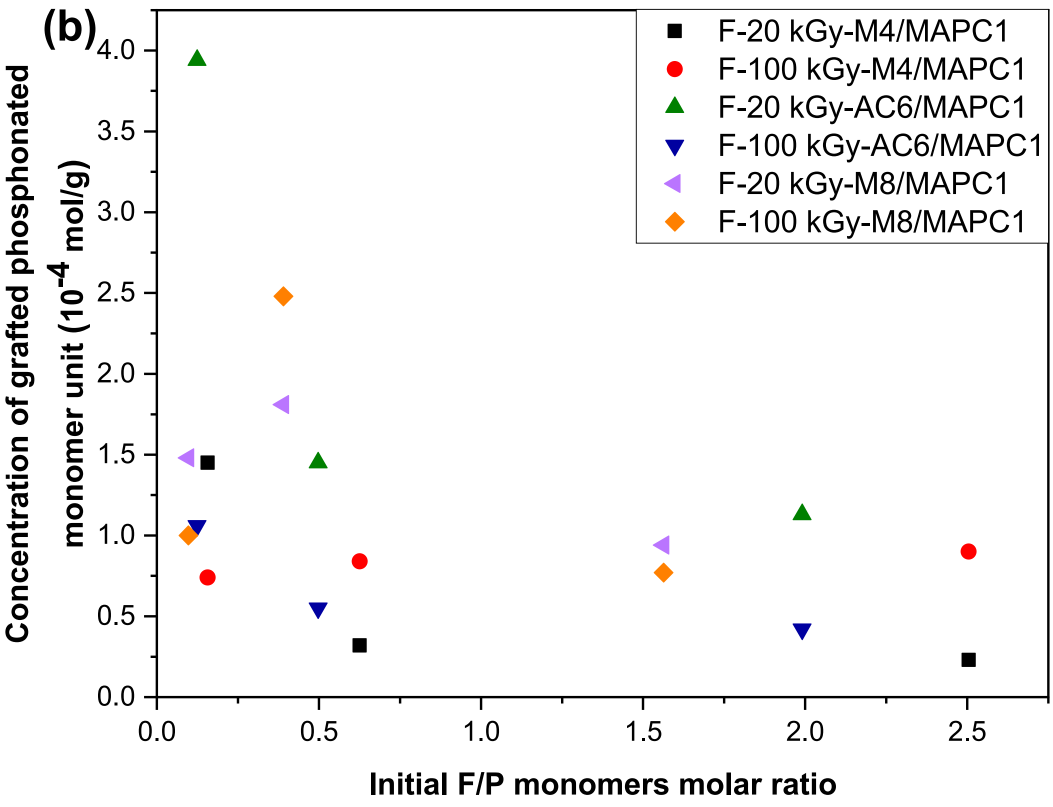
(b)
Concentration of grafted phosphonated monomer unit (10^{-4} mol/g)
Initial F/P monomers molar ratio
F-20 kGy-M4/MAPC1
F-100 kGy-M4/MAPC1
F-20 kGy-AC6/MAPC1
F-100 kGy-AC6/MAPC1
F-20 kGy-M8/MAPC1
F-100 kGy-M8/MAPC1

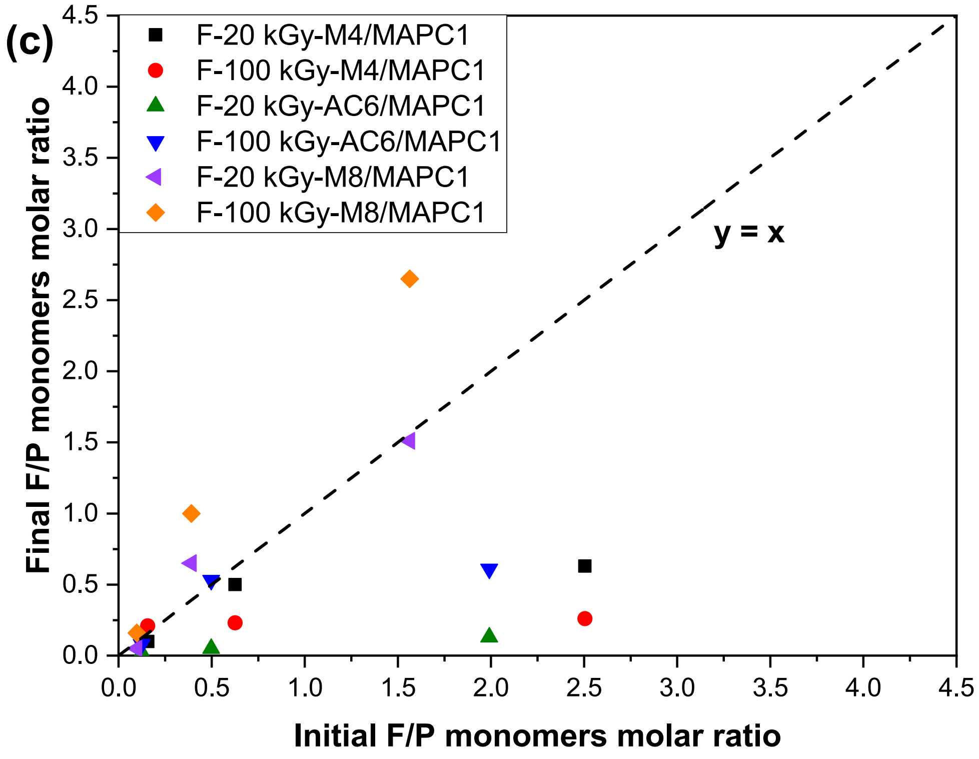

Figure S3 : Concentrations of grafted fluorinated (a) and phosphonated (b) monomers units and final F/P monomers molar ratio (c) versus the initial F/P monomer molar ratio.

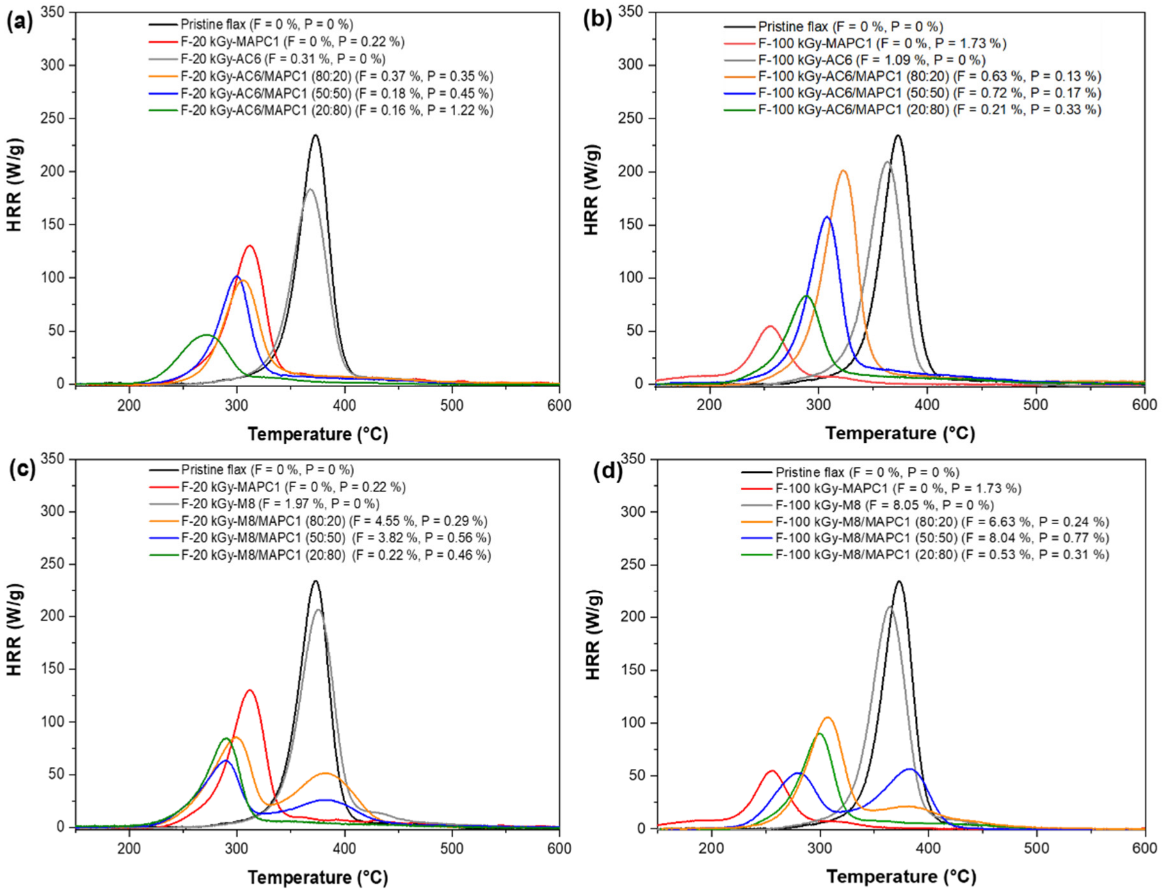

Figure S4 : HRR versus temperature curves in PCFC (anaerobic pyrolysis) of pre-irradiated flax fabrics at (a)(c) 20 kGy and at (b)(d) 100 kGy and treated with AC6/MAPC1 and M8/MAPC1 at various monomers ratio, respectively.

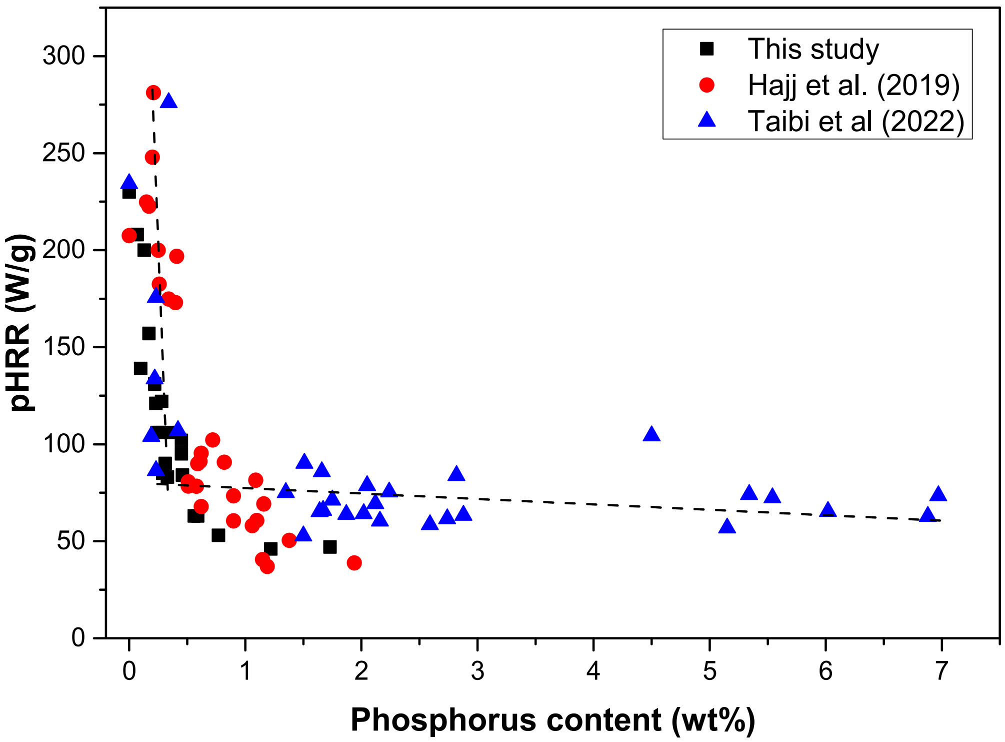

Figure S5 : PHRR versus phosphorus content in this study (combination of MAPC1 and fluorinated monomers) in comparison to previous works (only phosphonated monomers were grafted by pre-irradiation and simultaneous process) (dotted lines are guidelines for eyes).
